# Faecal immunochemical test for patients with ‘high-risk’ bowel symptoms: a large prospective cohort study and updated literature review

**DOI:** 10.1038/s41416-021-01653-x

**Published:** 2021-12-13

**Authors:** Helga E. Laszlo, Edward Seward, Ruth M. Ayling, Jennifer Lake, Aman Malhi, Clare Stephens, Kathy Pritchard-Jones, Donna Chung, Allan Hackshaw, Michael Machesney

**Affiliations:** 1 North Central London Cancer Alliance, 47 Wimpole Street, London, W1G 8SE UK; 2 University College London Hospitals NHS Foundation Trust, 235 Euston Road, London, NW1 2BU UK; 3 Barts Health NHS Trust—The Royal London Hospital, Whitechapel Road, London, E1 1BB UK; 4 Cancer Research UK & UCL Cancer Trials Centre, University College London, 90 Tottenham Court Road, London, W1T 4TJ UK; 5 North London Partners in Health and Care, 5 Pancras Square, London, N1C 4AG UK; 6 UCL GOS Institute of Child Health, University College London, 30 Guilford Street, London, WC1N 1EH UK; 7 North East London Cancer Alliance—Unex Tower, 5 Station Street, London, E15 1DA UK

**Keywords:** Colorectal cancer, Diagnostic markers

## Abstract

**Background:**

We evaluated whether faecal immunochemical testing (FIT) can rule out colorectal cancer (CRC) among patients presenting with ‘high-risk’ symptoms requiring definitive investigation.

**Methods:**

Three thousand five hundred and ninety-six symptomatic patients referred to the standard urgent CRC pathway were recruited in a multi-centre observational study. They completed FIT in addition to standard investigations. CRC miss rate (percentage of CRC cases with low quantitative faecal haemoglobin [f-Hb] measurement) and specificity (percentage of patients without cancer with low f-Hb) were calculated. We also provided an updated literature review.

**Results:**

Ninety patients had CRC. At f-Hb < 10 µg/g, the miss rate was 16.7% (specificity 80.1%). At f-Hb < 4 µg/g, the miss rate was 12.2% (specificity 73%), which became 3.3% if low FIT plus the absence of anaemia and abdominal pain were considered (specificity 51%). Within meta-analyses of 9 UK studies, the pooled miss rate was 7.2% (specificity 74%) for f-Hb < 4 µg/g.

**Discussion:**

FIT alone as a triage tool would miss an estimated 1 in 8 cases in our study (1 in 14 from meta-analysis), while many people without CRC could avoid investigations. FIT can focus secondary care diagnostic capacity on patients most at risk of CRC, but more work on safety netting is required before incorporating FIT triage into the urgent diagnostic pathway.

## Introduction

Colorectal cancer (CRC) is the second leading cause of cancer death [1]. Abdominal symptoms often result in people being referred for cancer investigations. In England for example, these symptoms are defined in a National Institute of Health and Care Excellence (NICE) guideline (NG12), and patients go through an urgent referral pathway and are seen by a specialist within 14 days [2]. In 2018/19 over 396,000 patients in England went through this pathway [3]. Most were investigated with colonoscopy, the gold standard for detecting CRC, high-risk adenoma (HRA) and inflammatory bowel disease (IBD). However, <8% of patients with high-risk symptoms have CRC [4].

Faecal haemoglobin (f-Hb) has a higher predictive value than symptoms of colorectal disease [5–7], with potential use as a simple test among symptomatic patients to facilitate early diagnosis of CRC, and thus improve cancer survival [8].

Several studies from multiple countries have examined the faecal immunochemical test (FIT), which quantifies f-Hb concentration, and its ability to reliably exclude CRC, HRA and IBD in asymptomatic (i.e. screening) populations [9–12], and symptomatic ‘lower-’ [13–19] and ‘higher-’ risk [8, 20–28] patient groups. A meta-analysis indicated that f-Hb has a higher test performance than the urgent referral pathway for all significant colorectal disease, and that referral using FIT to triage the use of colonoscopy could be more cost-effective than direct referral to colonoscopy [29]. NICE encouraged further research [30].

A recent large study of FIT, involving 9822 symptomatic patients due to have a colonoscopy, found that at the lowest level of detection (<2 µg/g), 3% of CRC cases were missed, while 65% of people without CRC were below this threshold [26]. Our paper has several purposes: to report the accuracy of FIT in another large contemporary study in the United Kingdom, to examine patient features that might improve accuracy and to perform an up-to-date literature review (including meta-analyses) of all studies to provide reliable estimates of the accuracy of FIT as a rule-out test.

## Methods

### Study design

We conducted a prospective multi-centre observational study (qFIT study), which recruited patients from 24 hospitals in England and 59 general practices in London between April 2017 and March 2019. Sites were invited through the National Institute for Health Research Clinical Research Network (NIHR CRN); (Supplementary Table 1). National ethical approval was granted. The study was conducted following the STARD 2015 guideline for diagnostic studies [31].

Adult patients presenting with abdominal symptoms and urgently referred for suspected CRC and all patients who met the NICE NG12 referral criteria were eligible. People under 16 years of age or those unable to understand instructions (including non-English speakers who did not have an interpreter) were not invited.

Patients on the urgent referral pathway were offered a FIT pack (containing the specimen collection device, a patient experience survey and an information booklet outlining the study purpose). They were asked to take a single sample at their next bowel movement, before completing bowel preparation for colonoscopy or other examination and post it without delay to a central laboratory (Supplementary Fig. 1). By returning the FIT specimen collection device and attached paperwork, the patient provided implied consent to participate. Patients were aware that the FIT result was for research purposes only and that they would not be informed of the result.

### Sample analysis

Faecal samples were taken with a specimen collection device and sent to the Clinical Biochemistry department at Barts Health NHS Trust by post. They were stored at 4 °C before analysis, which took place within 1 week of receipt and 2 weeks of sampling. The laboratory is accredited by the UK Accreditation Service to ISO 15189 standards. Analysis was performed using a single OC-Sensor™iO (Tokyo, Japan, Eiken Chemical Co., Ltd). Inter-run imprecision was assessed with quality control materials (Eiken) in each run. Coefficients of variation were 2.8% at 14 µg/g and 3.0% at 91 µg/g. External quality assurance was achieved via satisfactory performance in the relevant National External Quality Assurance Scheme. The lower limit of quantification was 4 µg/g, with a coefficient of variation of 7.7%. The upper analytical limit was 200 µg/g and samples with a concentration above this were not diluted and re-assayed, but reported as >200 µg/g. All laboratory analyses were performed blinded to patient characteristics and cancer outcomes.

### Outcomes

CRC and other bowel pathologies were diagnosed by the participating sites, as per standard practice (BSG guidelines 2010 [32]) and examination outcomes were confirmed to the study team. Clinicians were blinded to the FIT result. Hospitals also provided copies of endoscopy, radiology and histology reports, clinic letters and referral forms to the study team for further data extraction and quality control checks. Incomplete patient records were followed up until November 2019.

### Statistical considerations

We aimed to recruit at least 2200 patients to yield at least 80 CRC cases (assuming 3.5% prevalence, based on previous studies [20, 21]), which would have an acceptable error rate around a sensitivity of 89% based on prior studies (i.e. 95% confidence interval (CI) width of ±6.8%). Sensitivity, specificity, positive predictive value (PPV) and negative predictive value (NPV) were calculated as standard measures of test performance. For a cancer rule-out test, 100 minus sensitivity represents the proportion of CRC cases that would be missed by the test, and we label this the ‘CRC miss rate’, and refer to this instead of sensitivity. Both the CRC miss rate and specificity have a direct clinical consequence for patients and clinicians because they represent (respectively) harm (patients with CRC who would not have further investigations because their f-Hb was below some threshold, so their cancer would not be found at the time) and benefit (patients without CRC who might not be referred for invasive cancer investigations). Effective rule-out tests must have a low cancer miss rate and ideally high specificity. Spearman’s rank correlation and the Kruskal–Wallis test were used to assess associations between f-Hb and age, sex and ethnicity. Fisher’s exact test was used to assess differences in patient features between those with and without CRC cancer. STATA version 15 was used for all analyses [33].

### Meta-analyses

Several studies have now reported the performance of FIT as a rule-out test for CRC among symptomatic patients. Formal systematic reviews [7, 29] have already been conducted on this topic that followed the Preferred Reporting Items for Systematic Reviews (PRISMA) guidance and one was funded by the UK NIHR Health Technology Assessment programme. We, therefore, used those reports and updated the literature review to find the most recent studies, using the PRISMA guidance. We performed meta-analyses to see where our own study fits in with others, but importantly to provide robust estimates of test performance that avoid under- or over-estimating the cancer miss rate and specificity when based on individual studies. From PubMed we found a few studies published after the two previous reviews (up to 01 April 2021), using the following search terms: (“faecal immunochemical test” OR “fecal immunochemical test” OR “FIT” OR “faecal haemoglobin” OR “fecal hemoglobin”) AND (“colorectal cancer” OR “CRC” OR “bowel”) AND (“symptom*” OR “symptomatic”) AND (“sensitivit*”). AM reviewed and screened the titles and abstracts of articles retrieved in the search and determined eligibility by appraisal of full texts. As with the prior systematic reviews, we only included observational studies conducted in symptomatic patients and where the FIT result was not reported nor acted upon to manage patients.

Study characteristics and the CRC miss rate and specificity were extracted by AM and checked by AH. Risk of bias or issues with applicability were determined through assessments of each study’s methodology relating to patient selection, index tests, reference standards and patient flow and timing using the QUADAS-2 instrument for diagnostic studies. Exact 95% CIs were calculated, and pooled estimates of the two endpoints were obtained by a standard random effects model using DerSimonian and Laird’s method (in STATA) [33].

In one meta-analysis, we used data at the lowest available FIT cut-off using all studies, which was based on multiple f-Hb thresholds (to be consistent with previous reviews). However, we focussed on the contemporary studies conducted in the United Kingdom since 2015, which had identified at least ten CRC cases, and where all patients had been referred onto the same national NG12 cancer pathway and therefore used the same criteria for selecting patients. In these UK studies, the FIT result was not reported back to clinicians or patients and therefore not used to manage patients.

## Results

**Fig. 1:**
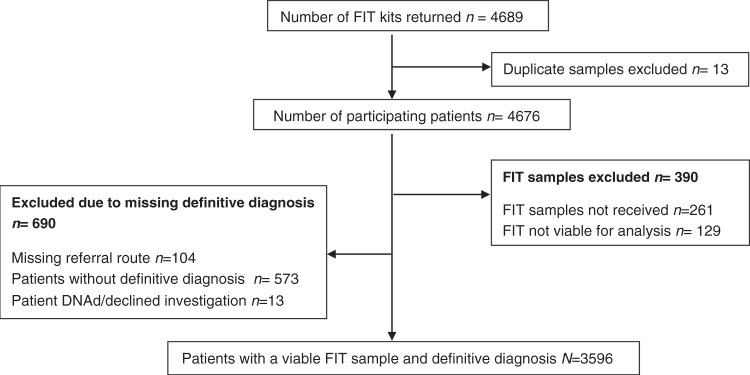
qFIT study flow diagram.

Within the 2-year accrual timeframe, around 8000 FIT packs were distributed across sites and returned from 4676 patients. Among these, 3596 patients provided an evaluable faecal sample and the outcomes after cancer investigations were reported to the coordinating centre (Fig. 1). We did not have ethics approval to collect demographic data on patients who declined to return a FIT.

The median age was 67 years (70% aged ≥60 years) and 53% were female (Table 1). The prevalence of the five most reported clinical features recorded on the urgent referral form was: change of bowel habit 1835 (51%), rectal bleeding 970 (27%), anaemia 684 (19%), abdominal pain 427 (11.9%) and weight loss 312 (8.7%). The first investigation recorded for each patient was colonoscopy (77.7%), CT colonography (14.2%) and flexible sigmoidoscopy (7.5%) (Supplementary Table 2).

**Table 1:** Patient characteristics.

Characteristics	Cancer outcome		
	Total, *N* = 3596	Any cancer, *N* = 97	No cancer found, *N* = 3499
Median age, years (range; IQR)	67 (19–99; 57–75)	71 (34–92; 63–78)	67 (19–99; 57–75)
Age group, years (%)			
<30	21 (0.6)	0 (0)	21 (0.6)
30–39	79 (2.2)	2 (2.1)	77 (2.2)
40–49	262 (7.3)	1 (1.0)	261 (7.5)
50–59	739 (20.6)	13 (13.4)	726 (20.7)
60–69	979 (27.2)	24 (24.7)	955 (27.3)
70–79	1016 (28.3)	41 (42.3)	975 (27.9)
80–89	476 (13.2)	14 (14.4)	462 (13.2)
90+	24 (0.7)	2 (2.1)	22 (0.6)
Missing data	N/A	N/A	N/A
Sex, number (%)			
Female	1911 (53.1)	40 (41.2)	1871 (53.5)
Male	1675 (46.6)	56 (57.7)	1619 (46.3)
Missing data	10 (0.3)	1 (1.0)	9 (0.3)
Age group of females, number (%)			
<30	12 (0.3)	0 (0)	12 (0.3)
30–49	182 (5.1)	2 (2.1)	180 (5.1)
50–79	1460 (40.6)	31 (32.0)	1429 (40.8)
80+	257 (7.1)	7 (7.2)	250 (7.1)
Missing data	N/A	N/A	N/A
Age group of males, number (%)			
<30	9 (0.3)	0 (0)	9 (0.25)
30–49	159 (4.4)	1 (1.0)	158 (4.5)
50–79	1267 (35.2)	46 (47.4)	1221 (34.9)
80+	240 (6.7)	9 (9.3)	231 (6.6)
Missing data	N/A	N/A	N/A
Ethnicity, number (%)			
Black/Black British	163 (4.5)	6 (6.2)	157 (4.5)
Asian/Asian British	220 (6.1)	1 (1.0)	219 (6.3)
Other Asian^a^	73 (2.0)	3 (3.1)	70 (2.0)
White	845 (23.5)	25 (25.8)	820 (23.4)
British mixed	645 (17.9)	18 (18.6)	627 (17.9)
Multiple/other	200 (5.6)	3 (3.1)	197 (5.6)
Missing data	1450 (40.3)	41 (42.3)	1409 (40.3)

^a^The ethnicity of ‘Other Asian’ consisted of those with Chinese ethnicity or Asian ethnicity other than Indian/Indian British, Pakistani/Pakistan British or Bangladeshi/Bangladeshi British.

The association between f-Hb and each of age, sex and ethnicity are shown in Supplementary Figs. 2–4. The correlation coefficients and differences were not clinically meaningful.

### Clinical outcomes and FIT performance

Ninety patients were diagnosed with CRC (2.5%) and seven patients had cancers of another type. The most common diagnoses among patients without cancer were diverticulosis 1101 (31.5%), polyps 805 (23%), adenomas 623 (17.8%), HRAs 61 (1.7%), haemorrhoids 526 (15%), and colitis 286 (8.2%). Table 2 summarises the performance of FIT at different f-Hb values. Data on IBD and advanced neoplasia will be reported separately.

**Table 2:** Test performance of FIT for colorectal cancer (CRC) at different f-Hb cut-offs.

Individuals with negative test results, i.e. below the specified f-Hb cut-off						Individuals with positive test results, i.e. at or above the specified f-Hb cut-off			
f-Hb cut-off (µg/g)	Not cancer (specificity)* (*n* = 3499) (TN)	Colorectal cancer*^#^ (*n* = 90) (FN)	Negative predictive value (%) (TN/TN + FN)	Risk of CRC among test negatives, (FN/TN + FN)	% of all patients beneath threshold (*N* = 3596)	f-Hb cut-off (µg/g)	Sensitivity (*n* = 90 colorectal cancers)* (TP)	False-positive rate* (*n* = 3499) (FP)	Positive predictive Value (%) (TP/TP + FP)
	No. (%)	No. (%)	%	No. per 1000	%		% (no.)	% (no.)	%
<4	2556 (73.0)	11 (12.2)	99.6	4.3	71.4	≥4	87.8 (79)	27.0 (943)	7.7
<6	2662 (76.1)	12 (13.3)	99.6	4.5	74.4	≥6	86.7 (78)	23.9 (837)	8.5
<10	2803 (80.1)	15 (16.7)	99.5	5.3	78.5	≥10	83.3 (75)	19.9 (696)	9.7
<20	2993 (85.5)	17 (18.9)	99.4	5.6	83.8	≥20	81.1 (73)	14.5 (506)	12.6
<50	3205 (91.6)	23 (25.6)	99.3	7.1	89.9	≥50	74.4 (67)	8.4 (294)	18.6
<80	3265 (93.3)	29 (32.2)	99.1	8.8	91.7	≥80	67.8 (61)	6.7 (234)	20.7
<100	3294 (94.1)	32 (35.6)	99.0	9.6	92.6	≥100	64.4 (58)	5.9 (205)	22.1
<120	3315 (94.7)	35 (38.9)	99.0	10.4	93.3	≥120	61.1 (55)	5.3 (184)	23.0
<150	3331 (95.2)	38 (42.2)	98.9	11.3	93.8	≥150	57.8 (52)	4.8 (168)	23.6
<200	3347 (95.7)	41 (45.6)	98.8	12.1	94.4	≥200	54.4 (49)	4.3 (152)	24.4

*Excludes 7 patients with cancer other than CRC.

^#^This is 100 minus sensitivity.

False-positive rate is the same as 100 minus specificity.

*TN* true negatives, *FN* False negatives, *TP* True positives, *FP* False positives.

At an f-Hb cut-off of <4 µg/g, 12.2% (95% CI 5.5–19.0) of CRC cases would be missed (1 in 8), but with a high specificity of 73% (95% CI 71.6–74.5). Using the cut-off of <10 µg/g, 1 in 6 CRC cases would be missed (16.7%, 95% CI 9.0–24.4) with a specificity of 80.1% (95% CI 78.9–81.4).

When considering FIT to detect CRC (Table 2), the PPV is higher using either ≥4 or ≥10 µg/g (7.7% or 9.7%, respectively) than with the urgent referral pathway (2.5%). Using f-Hb ≥10 µg/g, the sensitivity, false-positive rate and PPV were 83.3% (95% CI 75.6–91.0), 19.9% (95% CI 18.6–21.1) and 9.7% (95% CI 7.6–11.8), respectively. Increasing the threshold from ≥4 to ≥10 µg/g is associated with a modest decrease in sensitivity from 87.8 to 83.3%, and the false-positive rate decreases by 7.1 percentage points (from 27.0 to 19.9%).

In our study, there were seven patients with cancer other than CRC (Supplementary Table 3). Five of these had f-Hb ≥4 µg/g: anal, prostate and neuroendocrine tumour and two with lymphoma, so they would have been found at that threshold following cancer investigations after the referral. Two cancers had f-Hb <4 µg/g (neuroendocrine tumour and a lower rectal stromal tumour).

As we had found a relatively high CRC miss rate, we undertook exploratory (post hoc) analyses to investigate whether including clinical features could identify more patients with CRC. We examined the influence of patient symptoms when considered alongside the FIT test value. Among patients with f-Hb <10 µg/g, there was no association between the presence of CRC and either rectal bleeding or change in bowel habit (Table 3). However, CRC patients were more likely to present with abdominal pain and/or anaemia than non-cancer patients (66.7 vs. 29.4%, *p* = 0.003) (Table 3). Therefore, among all 90 CRC cases, only 3 would be missed because their f-Hb was <10 µg/g and they did not have anaemia nor abdominal pain (miss rate 1 in 30; 3.3% [3/90] and 95% CI 0–7.0). The corresponding specificity is 56% (1962/3499). Using the lowest cut-off (f-Hb<4 µg/g) and absence of anaemia and abdominal pain, the miss rate is the same as with 10 µg/g (i.e. 3.3%) and specificity 51% [1793/3499].

**Table 3:** Distribution of selected clinical features among individuals with and without CRC at f-Hb <10 µg/g (false negatives).

Clinical features	With CRC, *N* = 15 (%)	Without CRC, *N* = 2803 (%)	*P* value^a^
Anaemia	8 (53.3)	502 (17.9)	0.002
Abdominal pain	6 (40.0)	355 (12.7)	0.008
Either abdominal pain *or* anaemia	10 (66.7)	825 (29.4)	0.003
Both abdominal pain *and* anaemia	2 (13.3)	16 (0.6)	0.004
Neither pain nor anaemia	3 (20.0)	1962 (70.0)	<0.001
Change in bowel habit	9 (60.0)	1506 (53.7)	0.80
Rectal bleeding	4 (26.7)	691 (24.7)	0.77
Weight loss	1 (6.7)	246 (8.8)	>0.99
Family history of CRC	1 (6.7)	66 (2.4)	0.30
Abnormal imaging	1 (6.7)	33 (1.2)	0.17
Previous bowel cancer	1 (6.7)	0 (0)	-

^a^Fisher’s exact test.

At <4 µg/g, 3 CRC cases had neither abdominal pain nor anaemia, as did 1793 people without cancer (*p* < 0.001).

Patients with CRC and low FIT concentrations were also more likely to have multiple primary symptoms than non-cancer patients (Supplementary Table 4). Forty per cent of patients with CRC (6/15) who had f-Hb <10 µg/g presented with three or more of the symptoms listed in Table 3, but 68.7% of patients without CRC (1926/2803) had none or only one of these symptoms (*p* < 0.001). Similarly, 73.3% (11/15) of the CRC cases had two or three primary symptoms recorded (among anaemia, abdominal pain, rectal bleeding or abdominal pain), compared to only 18.6% (521/2803) of patients without CRC (*p* < 0.001).

Although the main purpose of using FIT as a triage tool is to help detect CRC, there is interest in finding people who have other bowel disorders such as IBD. Supplementary Table 5 shows FIT performance for detecting any cancer and IBD. At f-Hb <4 µg/g, 38.7% of all cases (all cancer types and IBD cases) could be missed. There were 213 cases of IBD in total, of which 50.2% had f-Hb <4 µg/g. Further details of FIT performance for pathology other than cancer will be reported separately.

### Literature review and meta-analyses

**Fig. 2:**
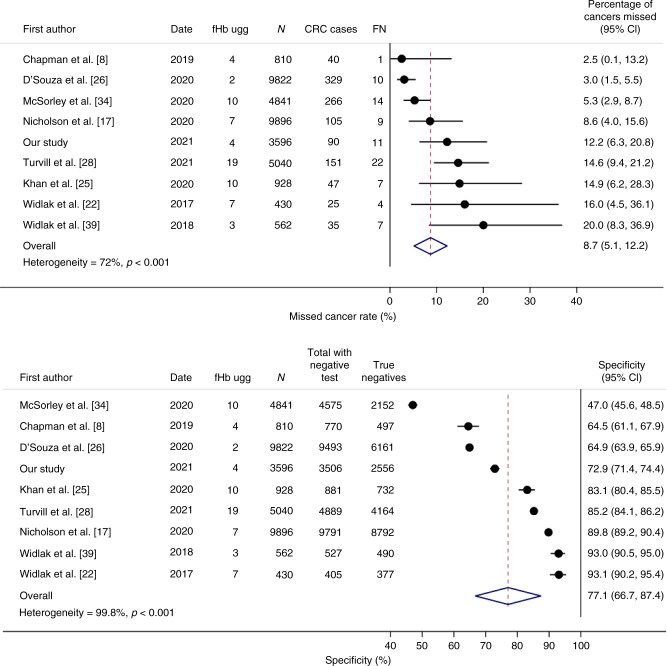
Nine studies were conducted in the United Kingdom (patients recruited between 2015 and 2020), in which all patients were referred using the NICE NG12 pathway. CRC miss rate (100 minus sensitivity), and specificity using the lowest limit of detection for f-Hb in each study. Excluding McSorley et al. (because it had a noticeably low specificity), the pooled specificity is 80.8% (95% CI 72.5–89.1). The OC-Sensor FIT assay was used in Chapman et al. [8] and our own study, and all others used HM-JACKarc; 1088 CRC cases. *N* total number of patients in each study, FN false negatives.

Sixty-two articles were identified, of which 20 were considered for inclusion in our meta-analyses [8, 13, 15, 17–22, 25–28, 34–42]. One recently conducted study from the United Kingdom was excluded because the FIT result was used to select patients for referral to investigations, rather than being sent in parallel with the national cancer diagnostic pathway (the included contemporary UK studies did not act on the FIT result) [19]. Supplementary Tables 6 and 7 show the study characteristics and performance measures of all studies. Acknowledging the differences in laboratory methods, FIT assays and local cut-offs, there was substantial variability in the miss rate (0–20%) and specificity (47–100%) when each study used its lowest limit of quantification (Supplementary Fig. 5). The variability remained when standardising to the same 10 µg/g cut-off (Supplemental Fig. 6). We, therefore, focussed on the nine contemporary studies (with at least ten CRC cases) conducted in the United Kingdom that only recruited patients between 2015 and 2020, and all via the same national cancer referral pathway (NICE NG12) to provide consistency. All nine studies (35,925 patients in total, including 1088 with CRC) used one of the FIT analysers recommended by NICE DG30 (OC-Sensor or HM-JACKarc) [30]. The pooled CRC miss rate is 8.7% and pooled specificity 77.1%, using the lowest limit of quantification reported for each study (Fig. 2), with corresponding estimates of 9.7 and 76.4% when the same 10 µg/g cut-off was used in all studies (Supplementary Fig. 7). Even among this apparently homogenous group of studies, variability in the accuracy of FIT is observed that is unlikely to all be due to differences in testing methodologies and analyser-specific cut-offs (the CRC miss rate ranged from 2.5 to 20%, and specificity 47.0 to 77.9%; Fig. 2). The heterogeneity is statistically significant.

Using the QUADAS-2 instrument for assessing study quality (risk of bias and applicability), all nine UK studies had a low risk of bias for almost all of the attributes (Supplementary Table 8). Therefore, subgroup analyses based on quality were not necessary.

In response to the variation in FIT cut-off values between the UK studies, we additionally focussed on the four studies that reported results for f-Hb <4 µg/g. The pooled CRC miss rate and specificity were 7.2% and 73.8% respectively (Supplementary Fig. 8), based on 14,790 patients in total and 494 CRC cases.

## Discussion

We report the results of a large study evaluating the use of FIT in people presenting with high-risk symptoms of bowel cancer, presenting the data in the context of a literature review and updated meta-analyses. The aim was to examine the contemporary evidence on whether FIT can as act as an effective ‘rule-out’ tool for CRC, allowing clinicians to triage patients with colorectal symptoms into a high-risk group warranting urgent investigation, and a lower risk group that could be given some reassurance and possibly further monitoring.

In our study, the lowest f-Hb threshold (<4 µg/g) would miss one in eight CRC cases, but with high specificity (73%), and at <10 µg/g, one in six CRC cases could be missed. We showed that f-Hb concentrations were not materially associated with age, sex or ethnicity as also seen in other studies of symptomatic patients [26], but unlike studies of asymptomatic people [43–45].

NPV is often reported as a measure of test performance for rule-out tests. However, very high NPVs can be due to having a large number of non-cancer patients in relation to a small number of CRC cases, particularly in small studies. The NPV in our study was 99.6% for f-Hb <4 µg/g, similar to other studies, but this masks that as many as 12% of cancers would be missed. High NPVs, therefore, give false reassurance about the effectiveness of the FIT test in ruling out CRC.

The nine studies recently conducted in the United Kingdom (Fig. 2) produce a pooled CRC miss rate of 8.7% and specificity of 77.1% (i.e. identifies people without cancer who might be able to avoid further investigations, unless there are other clinical indications for a referral, such as persistent symptoms, despite having a low f-Hb); the corresponding estimates were 7.2 and 74% in the four studies that used f-Hb <4 µg/g. Our own study values were approximately in the middle of the range across all studies (Fig. 2). Reasons for the variability in the CRC miss rate could include patient characteristics and the use of reference standards in addition to colonoscopy. However, when we focussed only on recent UK studies, reasons for the variability were not evident from the publications. All assay methods are recommended by NICE and because these UK studies recruited from patients on the NG12 referral pathway, it is unexpected that they would be fundamentally different. Among the four studies that reported data for f-Hb <4 µg/g including our own [8, 26, 39], patient characteristics were similar (Supplementary Table 6), and although the percentage of CRC cases found varied between 2.5 and 6.2%, these are consistent with the expected 2–8% using symptoms alone in the United Kingdom [46, 47]. The study by D’Souza et al. [26] was based on patients who had a colonoscopy, whereas our study included patients who had any investigation as part of the referral, which might partly explain the difference in the CRC miss rates and specificity. However, when we only examined patients who had a colonoscopy in our study, the CRC miss rate and specificity at f-Hb <4 µg/g were 10% and 74%, respectively, which was still different from 3 and 65% in D’Souza et al. [26]. It is likely, therefore, that the variability in FIT test performance we observed largely reflects the natural variability (chance) often seen in meta-analyses in medical research.

Our study used the OC-Sensor™iO, which is one of the recommended analysers in the NICE DG30 guidance [30], with a lower limit of quantification of 4 µg/g. NICE suggests that the three recommended analysers are comparable. However, there are differences in analytical performance [48] which may affect the generalisability of results between studies. The relevance of this is unknown, but it is a potential consideration. In our study, the 7.7% coefficient of variation at the lowest limit of detection appears to be higher than that expected at higher f-Hb thresholds, so it is possible that differences in test performance might be greater at low thresholds.

Our study is important because participants were not restricted by the type of examination performed on the urgent CRC pathway; they were drawn from a wide geography representing a diverse demographic across primary and secondary care. The cancer investigations were representative of pragmatic clinical practice; for example, the increased use of CT colonography for those over 80 years of age.

Our results suggest that patient symptoms (the presence of abdominal pain or anaemia at the time of referral) might be of use when considered alongside a negative FIT result. Abdominal pain is a common symptom, but in the context of a high-risk referral pathway, an obstructive cause may make it more worrisome. Other prospective studies [22, 42] and cohort studies [8, 49] have identified the same association of anaemia with FIT in the diagnosis of patients with CRC. Nevertheless, in our study, abdominal pain and anaemia were recorded once at the time of referral. If the practice were to change, it would need to be ascertained whether these clinical factors were persistent or not, and how this may influence the clinical decision to refer for cancer investigations.

Our study had a few limitations. First, 2.9% of our samples were unsuitable for FIT analysis. This is in line with previous discussions over stool self-sampling issues, such as delay in posting the sample back [50] or sampling the stool [51]. Only a single stool sample was requested from each patient, and Högberg et al. indicate that this could lead to missing one-tenth of symptomatic CRCs [52], compared to using three samples, while other studies did not find any significant improvement in test accuracy when two FITs were performed [23, 38]. Second, ethnicity data were not recorded for 40% of patients, although the percentage was similar between patients with and without cancer and thus may not have introduced bias. Third, a final clinical diagnosis was not recorded on the study case report forms for 696 patients during the study period. However, their exclusion would not have affected the results because their characteristics were similar to those included in the analyses (e.g. among the 696 patients, 79% had f-Hb <10 µg/g and 50% were male, with corresponding figures of 78 and 47% among those included in the analyses). Nevertheless, with 3596 patients (90 CRC cases) we clearly exceeded our target sample size of 2200 (80 cases). Fourth, we focus on the performance of FIT as a single rule-out test on its own, as specified in the study protocol and because it can be interpreted easily. Further statistical analyses are planned to attempt to develop a CRC prediction model using various parameters considered together (FIT, clinical symptoms and biochemical results such as haemoglobin).

Our study was performed in the context of considering largely symptom-based criteria for cancer referral. The current criteria for referral, which has an approximate 3% cancer rate, necessarily mean large numbers of patients referred will have normal test results, even allowing for other significant findings such as IBD or adenomas. Data from our study and the meta-analyses demonstrate that employing FIT as a triage tool can target unpleasant and potentially harmful investigations for patients who would most benefit. This represents a significant saving in healthcare resources and would address the problem of many referrals that are currently seen, which overburdens endoscopy and radiology departments without substantially improving the CRC diagnostic rate [53].

It is important to acknowledge that using FIT alone at any threshold can miss CRC cases, which would be found by the NG12 referral pathway, and that the magnitude of the miss rate varies between studies. Practical clinical use of FIT as a triage tool will clearly be hampered by concerns of both clinicians and patients about missed cancers (false-negative FIT). In the absence of a validated clinical risk score, a second clinical review could allow reassessment of the patient. Our study does not provide definitive guidance of how this safety netting should be conducted, or whether it should be in primary or secondary care. Clinicians and researchers should develop risk assessment tools for use in safety netting to minimise the number of missed CRCs. The (post hoc) analysis from our study that the presence of multiple clinical features, particularly anaemia or abdominal pain, appear to be more common in cancers with FIT below the threshold would suggest repeating a full blood count along with clinical reassessment would be appropriate. However, further studies are needed to confirm this. Demonstrating a decrease in haemoglobin, alongside additional clinical features, could be used to consider the patient for urgent referral. Further studies should evaluate the value of repeat FIT if symptoms persist.

NICE guidance in England currently recommends the use of FIT to triage ‘low-’ risk patients (those who do not meet the urgent CRC pathway referral criteria) presenting with lower abdominal symptoms in primary care [30], where an f-Hb concentration of ≥10 µg/g can be used to justify an urgent referral. We provide further evidence on the value of FIT in a higher-risk symptomatic group.

To conclude, we demonstrate that FIT is an effective triage tool with an estimated CRC miss rate of 1 in 14 from the meta-analysis (or 1 in 8 from our own study) for patients presenting with high-risk symptoms of CRC, while a substantial proportion of people without cancer (up to 3 in 4) might be able to avoid urgent investigations, depending on whether they are referred for other symptoms (e.g. clinical suspicion) regardless of their FIT level. FIT, therefore, has the ability to focus urgent diagnostic investigations on the patients most likely to have a CRC diagnosis. However, whilst effectively identifying patients for urgent cancer investigation, the use of FIT may also lead to many people without cancer avoiding investigations such as colonoscopy. This should improve the targeted allocation of healthcare resources. However, these benefits should not come at the expense of delaying the diagnosis of patients with CRC who present with symptoms, but have low FIT levels. Further work is needed to inform patients and clinicians of the necessary safety netting so that any FIT based referral guidance is as effective in case finding for CRC as the system it replaces.

## Supplementary information

Supplementary appendix

## Data Availability

Anonymised data will be shared on reasonable request to the corresponding author.
