# Supplementary material for: Faecal immunochemical test for patients with ‘high-risk’ bowel symptoms: a large prospective cohort study and updated literature review
Source: Br J Cancer. 2021 Dec 13;126(5):736–43. doi: 10.1038/s41416-021-01653-x (PMC8888593; doi:10.1038/s41416-021-01653-x)
Supplement: Supplementary file 1 — Supplementary appendix [file 41416_2021_1653_MOESM1_ESM.docx]

**Supplementary tables and figures**

**Supplementary Table 1.** List of participating sites in the qFIT study.

| **Primary care sites** | **CCG** |
| --- | --- |
| St Albans Surgery (formerly Dr Mohan and Associates) | Barking & Dagenham |
| Penshurst Gardens Surgery | Barnet |
| The Old Court House Surgery | Barnet |
| The Speedwell Practice | Barnet |
| Millway Medical Practice | Barnet |
| Oak Lodge Medical Centre | Barnet |
| PHGH Doctors | Barnet |
| Langdon Hills Medical Centre (Dr Salako & Partners) | Basildon and Brentwood |
| The Tile House Surgery | Basildon and Brentwood |
| Holborn Medical Centre | Camden |
| West Hampstead Medical Centre | Camden |
| Caversham Group Practice | Camden |
| Hampstead Group Practice | Camden |
| Keats Group Practice | Camden |
| Museum Practice | Camden |
| Parliament Hill Medical Centre | Camden |
| Prince of Wales Medical Centre | Camden |
| St Philips Medical Centre | Camden |
| Swiss Cottage Surgery | Camden |
| Elsdale Street Surgery | City & Hackney |
| The Well Street Surgery | City & Hackney |
| Woodberry Practice | Enfield |
| Brick Lane Surgery | Enfield |
| Eagle House Surgery | Enfield |
| East Enfield Medical Practice | Enfield |
| Morecambe Surgery | Enfield |
| Somerset Gardens Family Health Centre | Haringey |
| The Muswell Hill Practice | Haringey |
| Maylands Health Care | Havering |
| Petersfield Surgery | Havering |
| Bridgewater Surgeries | Herts Valley |
| Davenport House Surgery | Herts Valley |
| Harvey Group Practice | Herts Valley |
| Parkwood Surgery | Herts Valley |
| Fernville Surgery | Herts Valley |
| Manor View Practice | Herts Valley |
| City Road Medical Centre | Islington |
| The Miller Practice | Islington |
| Killick Street Health Centre | Islington |
| St Peter's Street Medical Practice | Islington |
| The Rise Group Practice | Islington |
| The Tufnell Surgery (now Junction Medical Practice) | Islington |
| Bute House Medical Centre | Luton |
| Beauchamp House Surgery | Mid Essex |
| Lord Lister Health Centre (Driver & Partners) | Newham |
| Wood Grange Medical Centre | Newham |
| Ilford Medical Centre | Redbridge |
| Wanstead Place Surgery | Redbridge |
| Glebelands Practice | Redbridge |
| Ilford Lane Surgery | Redbridge |
| The Willows Medical Practice | Redbridge |
| Chrisp Street Health Centre | Tower Hamlets |
| Limehouse Practice | Tower Hamlets |
| St Stephens Health Centre | Tower Hamlets |
| Ongar Health Centre | West Essex |
| Kings Medical Centre | West Essex |
| Lister Medical Centre | West Essex |
| Loughton Surgery | West Essex |

| **Secondary care sites** |
| --- |
| Barnet Hospital |
| Burnley General Hospital |
| Bury General Hospital |
| Chase Farm Hospital |
| Homerton University Hospital |
| King George Hospital |
| Manchester UFT (Oxford Road Campus) |
| Newham Hospital |
| North Manchester General Hospital |
| North Middlesex University Hospital |
| Queen's Hospital |
| Rochdale Infirmary |
| Royal Blackburn Hospital |
| Royal Free Hospital |
| Stockport NHS foundation trust |
| Tameside & Glossop Hospital |
| The Princess Alexandra Hospital |
| The Royal London Hospital |
| The Royal Oldham Hospital |
| The Whittington Hospital |
| University College London Hospital |
| West Hertfordshire Hospital NHS Trust |
| Whipps Cross University Hospital |
| Wythenshawe Hospital |

**Supplementary Table 2.** First type of exam by age group (in patients with a definitive diagnosis and a valid FIT) N=3585.

| Type of exam | Age group | Number of patients that had exam (N; %) | Total N=3596 |
| --- | --- | --- | --- |
| Colonoscopy | <30 | 17 (0.6) | 2794 |
|  | 30-49 | 300 (10.7) |  |
|  | 50-79 | 2239 (80.1) |  |
|  | 80+ | 238 (8.5) |  |
| CT Colonography | <30 | 0 (0) | 511 |
|  | 30-49 | 11 (2.2) |  |
|  | 50-79 | 312 (61.1) |  |
|  | 80+ | 188 (36.8) |  |
| Flexible Sigmoidoscopy | <30 | 4 (1.5) | 268 |
|  | 30-49 | 29 (10.8) |  |
|  | 50-79 | 168 (62.7) |  |
|  | 80+ | 67 (25.0) |  |
| CT Scan | <30 | 0 (0) | 5 |
|  | 30-49 | 0 (0) |  |
|  | 50-79 | 3 (60.0) |  |
|  | 80+ | 2 (40.0) |  |
| Rigid Sigmoidoscopy | <30 | 0 (0) | 1 |
|  | 30-49 | 0 (0) |  |
|  | 50-79 | 1 (100) |  |
|  | 80+ | 0 (0) |  |
| Other | <30 | 0 (0) | 4 |
|  | 30-49 | 1 (25.0) |  |
|  | 50-79 | 2 (50.0) |  |
|  | 80+ | 1 (25.0) |  |
| Not Applicable | <30 | 0 (0) | 2 |
|  | 30-49 | 0 (0) |  |
|  | 50-79 | 1 (50.0) |  |
|  | 80+ | 1 (50.0) |  |
| Missing Data | <30 | 0 (0) | 11 |
|  | 30-49 | 0 (0) |  |
|  | 50-79 | 8 (72.7) |  |
|  | 80+ | 3 (27.3) |  |

## **Supplementary Table 3.** Patients found to have cancers other than colorectal adenocarcinoma and their respective other diagnoses, symptoms and FIT concentrations (µg/g).

| No | Sex | Age | Type of Cancer | Other diagnoses | Symptoms | f-Hb (µg/g) |
| --- | --- | --- | --- | --- | --- | --- |
| 1 | Male | 84 | Anal cancer | N/A | Rectal bleeding; Change in bowel habit | 6.8 |
| 2 | Male | 77 | Diffuse B-cell lymphoma | Diverticulosis | Abnormal imaging | 165 |
| 3 | Male | 52 | Lower rectal Gastrointestinal Stromal Tumour | N/A | N/A | <4 |
| 4 | Male | 79 | Lymphoma | N/A | Change in bowel habit; Abdominal mass | 8 |
| 5 | Male | 58 | Prostate cancer | Polyps; Diverticulosis | Rectal bleeding | >200 |
| 6 | Male | 75 | Neuroendocrine carcinoma | Polyps | Change in bowel habit | >200 |
| 7 | Male | 69 | Neuroendocrine carcinoma | Colitis; Diverticulosis; Haemorrhoids | Change in bowel habit; Weight loss | <4 |

**Supplementary Table 4a.** Comparison of the number of primary clinical features (i.e. those with anaemia, abdominal pain, rectal bleeding or change in bowel habit) in patients with and without colorectal cancer who had f-Hb <10µg/g. No patient had all 4 features.

| Number of clinical features | With Colorectal cancer N=15 (%) | Without Colorectal cancer N=2803 (%) |
| --- | --- | --- |
| 0* | 1 (6.7) | 302 (10.8) |
| 1 | 3 (20.0) | 1980 (70.6) |
| 2 | 9 (60.0) | 489 (17.5) |
| 3 | 2 (13.3) | 32 (1.1) |

Fisher’s Exact test p<0.001

*these patients were referred on the urgent symptomatic pathway, but they did not have anaemia, abdominal pain, rectal bleeding or abdominal pain recorded

**Supplementary Table 4b.** Comparison of the number of clinical features (listed in Table 3 of the main paper) in patients with and without colorectal cancer who had f-Hb <10µg/g.

| Number of clinical features | With Colorectal cancer N=15 (%) | Without Colorectal cancer N=2803 (%) |
| --- | --- | --- |
| 0* | 1 (6.7) | 108 (3.8) |
| 1 | 3 (20.0) | 1818 (64.9) |
| 2 | 5 (33.3) | 686 (24.5) |
| 3 | 6 (40.0) | 191 (6.8) |

Fisher’s Exact test p<0.001

*these patients were referred on the urgent symptomatic pathway, but they did not have any of the features listed in Table 3 recorded

**Supplementary Table 5. Test performance of FIT for any cancer or Inflammatory Bowel Disease (IBD) at different f-Hb cut offs.**

| Individuals with negative test results, i.e. below the specified f-Hb cut-off | | | | | | |
| --- | --- | --- | --- | --- | --- | --- |
| f-Hb cut off (µg/g) | Not Cancer or IBD (specificity)* (n=3286)  (TN) | Any Cancer or IBD*# (n=310)  (FN) | Negative Predictive Value (%)  (TN/TN+FN) | Risk of Cancer or IBD among test negatives,  (FN/TN+FN) | % of all patients beneath threshold (N=3596) | IBD only  (n=213)  (FN) |
|  | No. (%) | No. (%) | % | No. per 1000 | % | No. (%) |
| <4 | 2449 (74.5) | 120 (38.7) | 95.3 | 46.7 | 71.4 | 107 (50.2) |
| <6 | 2548 (77.5) | 128 (41.3) | 95.2 | 47.8 | 74.4 | 114 (53.5) |
| <10 | 2677 (81.5) | 145 (46.8) | 94.9 | 51.4 | 78.5 | 126 (59.1) |
| <20 | 2854 (86.9) | 160 (51.6) | 94.7 | 53.1 | 83.8 | 139 (65.3) |
| <50 | 3050 (92.8) | 182 (58.7) | 94.4 | 56.3 | 89.9 | 155 (72.8) |
| <80 | 3106 (94.5) | 192 (61.9) | 94.2 | 58.2 | 91.7 | 159 (74.7) |
| <100 | 3129 (95.2) | 201 (64.8) | 94.0 | 60.4 | 92.6 | 165 (78.9) |
| <120 | 3144 (95.7) | 210 (67.7) | 93.7 | 62.6 | 93.3 | 171 (80.3) |
| <150 | 3160 (96.2) | 213 (68.7) | 93.7 | 63.1 | 93.8 | 171 (80.3) |
| <200 | 3171 (96.5) | 222 (71.6) | 93.5 | 65.4 | 94.4 | 176 (82.6) |

*excludes 7 patients with cancer other than CRC

#this is 100 minus sensitivity

Sensitivity at each threshold can be calculated by subtracting FN from column total divided by the column total

TN:true negatives FN:false negatives TP: true positives FP:false positives

# **Supplementary Table 6. Key Characteristics of studies assessing the diagnostic performance of FIT in detecting colorectal cancer (CRC) in a symptomatic population**

| **Study first author [reference]** | **Country** | **Period of recruitment** | **Average Age**  **(Mean or Median)** | **Gender** | | **f-HB** |
| --- | --- | --- | --- | --- | --- | --- |
|  |  |  |  | Male (%) | Female (%) | <10 μg/g (%)^a^ |
| **Our study** | UK | 2017-2019 | 65.7 | 46.6 | 53.1 | 78.4 |
| Turvill 2021 [28] | UK | 2018-2019 | 67.4 | 44.7 | 55.3 | - |
| D’Souza 2020 [26] | UK | 2017-2019 | 64.0 | 45.1 | 54.9 | 81.0 |
| McSorley *s*2020 [34] | UK | 2015-2019 | 60-66 | 43.2-47.3 | 52.7-56.4 | 44.7 |
| Navarro 2020 [35] | Spain | 2016-2018 | 58.5 | 44.3 | 55.7 | 71.7 |
| Mattar 2020 [36] | Brazil | 2015-2016 | 56.3 | 33.9 | 66.1 | 84.3 |
| Hogberg - Diaquick FOB  2020 [37] | Sweden | 2015 | 65 | 40.6 | 59.4 | - |
| Hogberg - Actim faecal blood  2020 [37] | Sweden | 2015 | 62 | 39.3 | 60.7 | - |
| Hogberg - Chemtrue FOB  2020 [37] | Sweden | 2015 | 67 | 37.8 | 62.2 | - |
| Hogberg - Analyz FOB  2020 [37] | Sweden | 2015 | 66 | 38.4 | 61.6 | - |
| Nicholson 2020 [17] | UK | 2017-2020 | 60 | 41.4 | 58.6 | 90.5 |
| Tsapournas 2020 [38] | Sweden | 2013-2017 | 65 | 42.1 | 57.9 | 73.6 |
| D’Souza 2020 [15] | UK | 2016-2017 | 60.6 | 48.6 | 51.4 | 81.7 |
| Chapman 2019 [8] | UK | 2016-2017 | 71.7 | 44.3 | 55.7 | 70.5 |
| Nicholson 2019 [13] | UK | 2016 | 58 | 43.0 | 57.0 | 88.2 |
| Khan 2020 [25] | UK | 2017-2018 | 72 | 40.5 | 59.5 | 79.6 |
| Ayling 2020 [18] | UK | 2019 | 60 | 44.3 | 55.7 | 51.2 |
| Widlak 2018 [39] | UK | - | 68 | 51.0 | 49.0 | - |
| Hogberg  2017 [42] | Sweden | 2013-2014 | 63 | 35.4 | 64.6 | - |
| Widlak 2017 [22] | UK | 2015-2016 | 67 | 49.0 | 51.0 | - |
| Mowat 2016 [20] | UK | 2013-2014 | 64 | 45.3 | 54.7 | 76.0 |
| Godber 2016 [21] | UK | 2013 | 59 | 44.8 | 55.2 | 75.0 |
| Rodriguez-Alonso 2015 [27]^b^ | Spain | 2011-2012 | - | 46.8 | 53.2 | 77.6 |
| Terhaar sive Droste [41] | Netherlands | 2006-2009 | 61.8 | 46.2 | 53.8 | 82.8 |

^a^The percentage below f-Hb 10 μg/g was obtained from studies where this information was provided

^b^ Provided proportions for age groups: <40 years 7.1%; 41-50 years 13.0%; 51-60 years 22.4%; 61-70 years 29.1%; >70 years 28.4%

# **Supplementary Table 7. Summary of studies assessing the diagnostic performance of FIT in detecting colorectal cancer (CRC) in a symptomatic population**

| Study  First author [reference] | Total (N) | CRC  Prevalence % (cases) | f-Hb cut-off (µg/g )^1^ | False Negatives (Missed cancers) | True Negatives | Sensitivity (%) | Specificity (%) | PPV (%) | NPV (%) |
| --- | --- | --- | --- | --- | --- | --- | --- | --- | --- |
| **This study** | 3596 | 2.5 (90) | 4 | 11 | 2556 | 87.8 | 73.0 | 7.7 | 99.6 |
|  |  |  | 10 | 15 | 2803 | 83.3 | 79.9 | 9.7 | 99.5 |
| Turvill [28] | 5040 | 3.0 (151) | 19 | 22 | 4164 | 85.4 | 85.2 | 15.1 | 99.5 |
|  |  |  |  |  |  |  |  |  |  |
| D’Souza [26] | 9822 | 3.3 (329) | 2 | 10 | 6161 | 97.0 | 64.9 | 8.7 | 99.8 |
|  |  |  | 10 | 30 | 7927 | 90.9 | 83.5 | 16.1 | 99.6 |
| McSorley [34] | 4841 | 5.5 (266) | 10 | 14 | 2152 | 94.7 | 47.0 | 9.4 | 99.4 |
| Navarro [35] | 727 | 5.0 (36) | 10 | 2 | 519 | 94.4 | 75.1 | 16.5 | 99.6 |
|  |  |  | 20 | 3 | 536 | 91.7 | 77.6 | 17.6 | 99.4 |
| Mattar [36] | 172 | 3.5 (6) | 10 | 1 | 144 | 83.3 | 86.7 | 17.8 | 82.2 |
| Hogberg - Diaquick FOB  [37] | 1751 | 2.2 (38) | 5 | 7 | 1361 | 81.6 | 79.5 | 8.1 | 99.5 |
| Hogberg :Actim faecal blood [37] | 4232 | 1.9 (79) | 25-50 | 2 | 2923 | 97.5 | 70.4 | 5.9 | 99.9 |
| Hogberg: Chemtrue FOB  (2020) [37] | 1756 | 1.7 (29) | 40 ng/ml | 0 | 1134 | 100.0 | 65.7 | 4.7 | 100.0 |
| Hogberg: Analyz FOB [37] | 7889 | 2 (158) | 2 | 18 | 5641 | 88.6 | 73.0 | 6.3 | 99.7 |
| Nicholson [17] | 9896 | 1.1 (105) | 7 | 9 | 8792 | 91.4 | 89.8 | 8.7 | 99.9 |
|  |  |  | 10 | 10 | 8943 | 90.5 | 91.3 | 10.1 | 99.9 |
| Tsapournas  [38] | 242 | 5 (13) | 10 | 1 | 177 | 92.3 | 77.3 | 18.8 | 99.4 |
| D’Souza  [15] | 218 | 3.7 (8) | 2 | 0 | 210 | 100.0 | 100.0 | 100.0 | 100.0 |
|  |  |  | 10 | 1 | 177 | 87.5 | 84.3 | 14.6 | 99.4 |
| Chapman  [8] | 810 | 4.9 (40) | 4 | 1 | 497 | 97.5 | 64.5 | 12.5 | 99.8 |
|  |  |  | 10 | 5 | 566 | 87.5 | 73.5 | 14.6 | 99.1 |
| Nicholson  [10] | 238 | 2.9 (7) | 7 | 1 | 206 | 85.7 | 89.2 | 19.4 | 99.5 |
|  |  |  | 10 | 1 | 209 | 85.7 | 90.5 | 21.4 | 99.5 |
| Khan [25] | 928 | 5.1 (47) | 10 | 7 | 732 | 85.1 | 83.5 | 22.6 | 99.0 |
| Ayling [18] | 217 | 3.7 (8) | 10 | 1 | 110 | 87.5 | 52.6 | 6.6 | 99.1 |
| Widlak [39] | 562 | 6.2 (35) | 3 | 7 | 490 | 80.0 | 93.0 | 44.0 | 99.0 |
| Hogberg [42] | 373 | 2.1 (8) | 20 | 0 | 188 | 100.0 | 51.5 | 4.3 | 100.0 |
| Widlak [22] | 430 | 5.8 (25) | 7 | 4 | 377 | 84.0 | 93.0 | 44.0 | 99.0 |
| Mowat [20] | 755 | 3.7 (28) | 10 | 3 | 571 | 89.3 | 79.1 | 14.1 | 99.5 |
| Godber [21] | 507 | 2.2 (11) | 10 | 0 | 380 | 100.0 | 76.6 | 8.7 | 100 |
| Rodriguez-Alonso [27] | 1003 | 3.0 (30) | 10 | 1 | 777 | 96.7 | 79.9 | 12.9 | 99.9 |
| Terhaar sive Droste [41] | 2058 | 5.4 (112) | 10 | 10 | 1693 | 91.1 | 87.0 | 28.7 | 99.4 |

^1^Cut-offs: less than the cut-off value was used for determining false negatives, true negatives, specificity, Negative predictive value (NPV); those more than the cut-off value was used for determining sensitivity and PPV

# **Supplementary Table 8**. Quality assessment of the 9 UK studies in the meta-analysis using the QUADAS-2 instrument

| **Bias** | | | |  | **Applicability** | | |
| --- | --- | --- | --- | --- | --- | --- | --- |
|  | **PATIENT SELECTION** | **INDEX TEST** | **REFERENCE STANDARD** | **FLOW AND TIMING** | **PATIENT SELECTION** | **INDEX TEST** | **REFERENCE STANDARD** |
| Our study | Low | Low | Low | Low^1^ | Low | Low | Low |
| Chapman *et al.* [8] | Low | Low | Low | Low | High | Low | Low |
| D'Souza *et al*. [26] | Low | Low | Low | Low | Low | Low | Low |
| McSorley *et al.* [34] | Low | Low | Low | Low | Low | Low | Low |
| Nicholson *et al.* [17] | Low | Low | Low | Low | Low | Low | Low |
| Turvill *et al.* [28] | Low | Low | Low | Low | Low | Low | Low |
| Khan *et al.* [25] | Low | Low | Low | Low | Low | Low | Low |
| Widlak  *et al.* [22] | Low | Low | Low | Low | Low | Low | Low |
| Widlak et al. [39] | Low | Low | Low | Low | Low | Low | Low |

^1^There are 3 attributes under ‘flow and timing’ and our study had low bias for two of these (i.e. short time interval between the index text and the reference standard test; and all patients received the same reference standard). However, our study did not meet the third feature (all patients should be included in the analysis), because 696 patients did not have a recorded cancer diagnosis reported to the study group. Nevertheless, these 696 seemed to be a random subset of all patients because there were no systematic differences between those with a missing cancer diagnosis and those with a cancer diagnosis; so there is likely to be no bias.

Urgent referral for suspected CRC

FIT pack offered by participating GP

Patient booked into hospital clinic (outpatient or telephone triage (straight-to-test))

Diagnostic tests according to patient suitability

Patient visits GP with abdominal symptoms

Sample & paperwork sent back by patient to lab

Hospital records diagnostic outcome

FIT pack offered by participating hospital

Results sent to qFIT study team

Consultant Clinic/Clinical nurse specialists telephone clinic

Endoscopist triage and book

FIT analysed in lab

Patient collects sample at home

Patient continues diagnosis-appropriate pathway

Data extraction

Data quality check

**Supplementary Figure 1.** Flow diagram for the study processes.

**Supplementary Figure 2**. Association between the f-Hb concentration and age among 3499 patients without cancer (upper figure) and 97 with cancer (lower figure). Due to the large sample size of non-cancers, the clinically insignificant correlation of 0.097 is statistically significant.


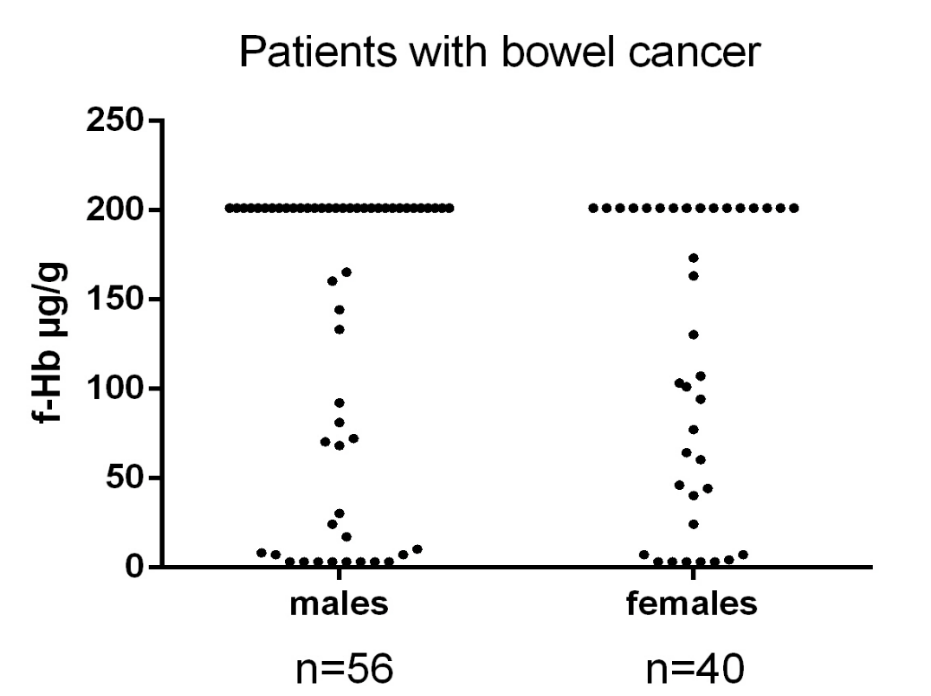


Kruskal Wallis test p=0.45


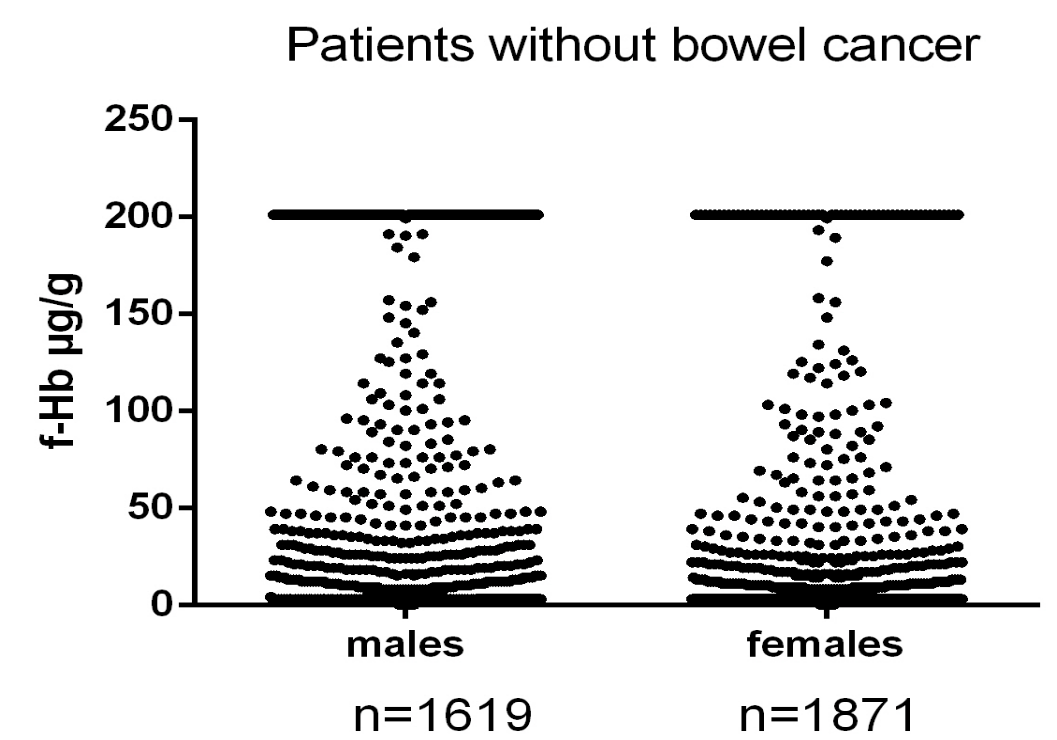


Kruskal Wallis test p<0.001

**Supplementary Figure 3**. Association between the f-Hb concentration and sex among 3490 patients without cancer (upper figure) and 97 with cancer (lower figure). In those without cancer the median f-Hb is <4 ug/g (males) and <4 ug/g (females), of which 76% (females) and 70% (males) had f-Hb <4 ug/g; this clinically insignificant difference is statistically significant due to the very large sample size.


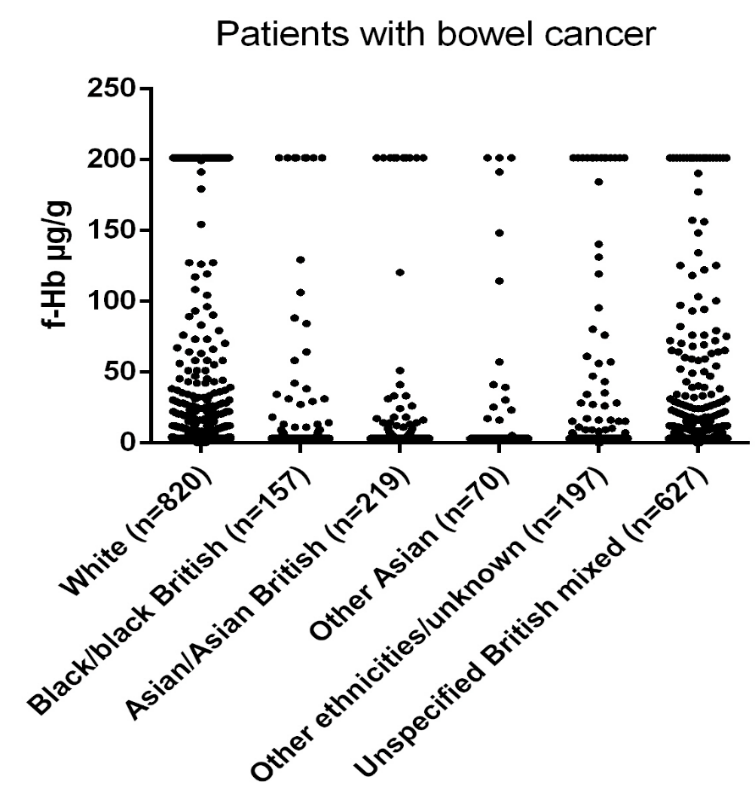


Kruskal Wallis test p=0.87


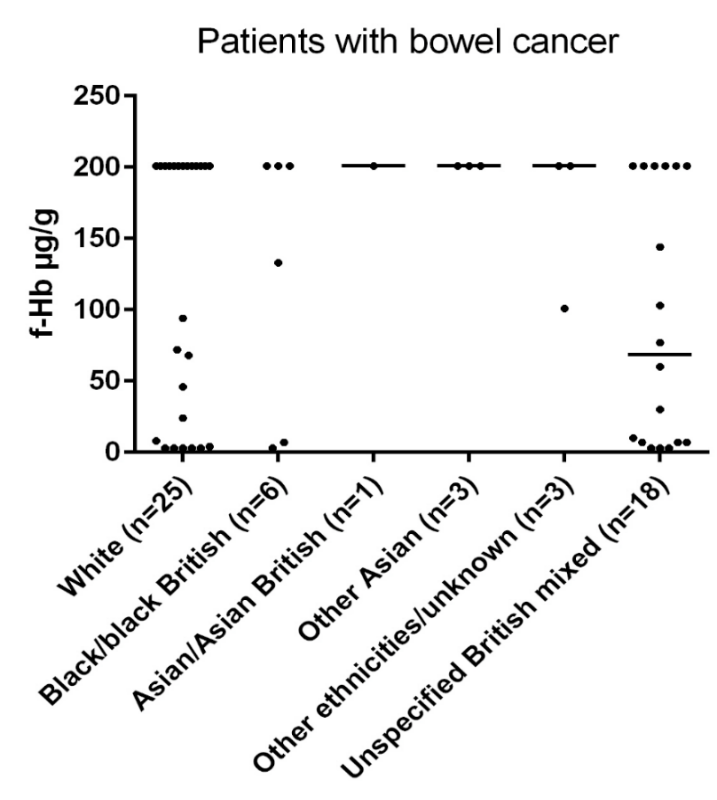


Kruskal Wallis test p=0.43

**Supplementary Figure 4**. Association between the f-Hb concentration and ethnicity among 2090 patients without cancer (upper figure) and 56 with cancer (lower figure).


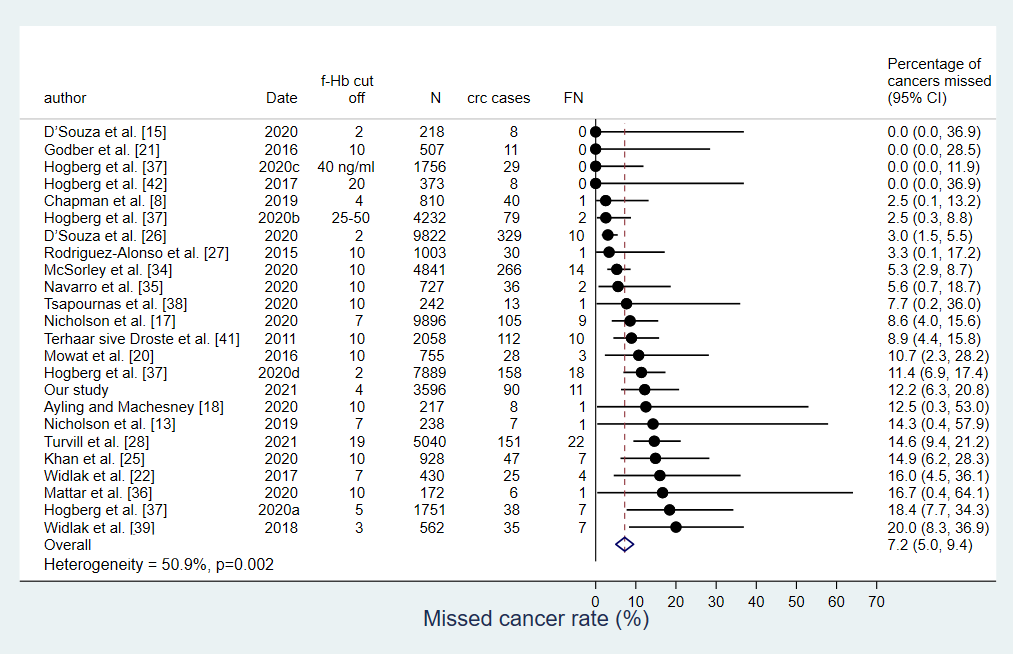

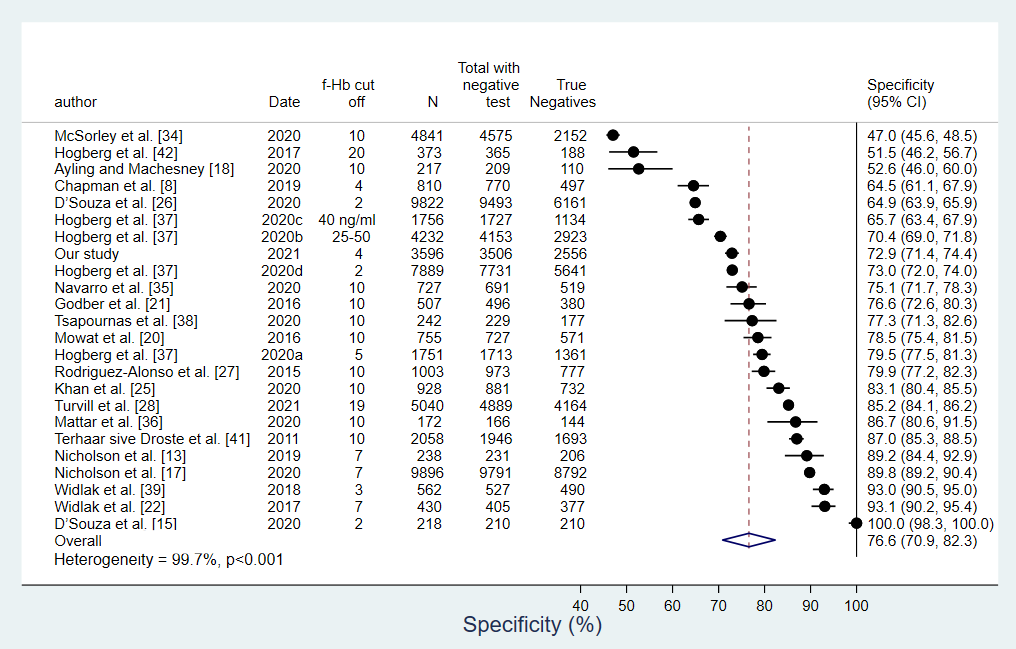


**Supplementary Figure 5**. Forest Plots of the CRC miss rate and specificity at the lowest cut-off of f-HB used in each study: 24 Studies; 1659 CRC cases (N=total; FN = false negatives, ie missed cancers)


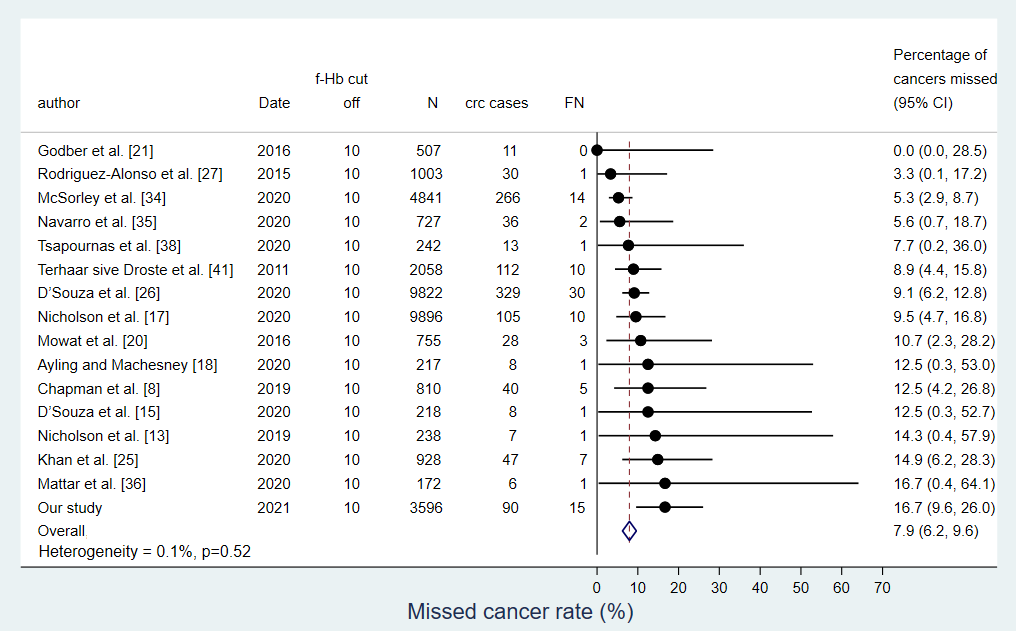

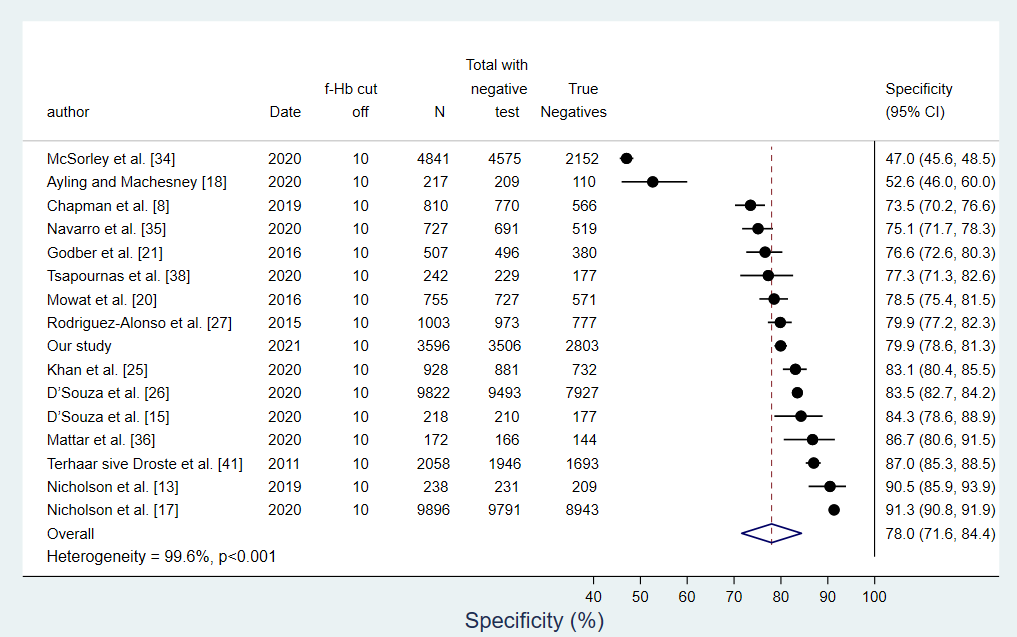


**Supplementary Figure 6**. Forest Plots of the CRC miss rate and specificity at a f-Hb cut-off of <10ug/g for all studies. 16 Studies; 1136 CRC cases (N=total; FN = false negatives, ie missed cancers). Excluding McSorley et al (low specificity), the pooled specificity is 80.4% (95% CI 77.0-83.7%)


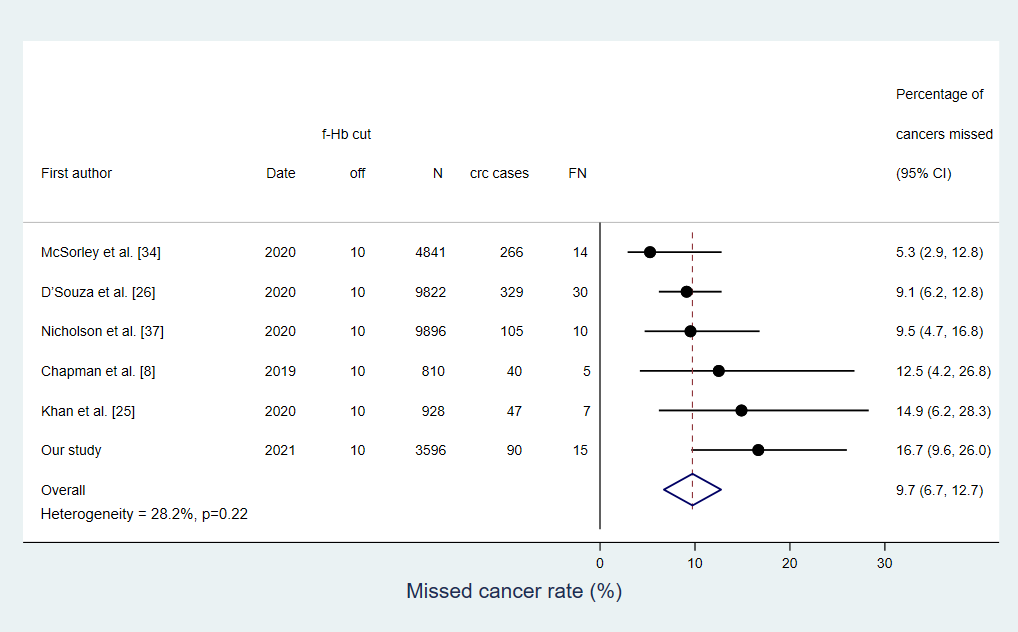

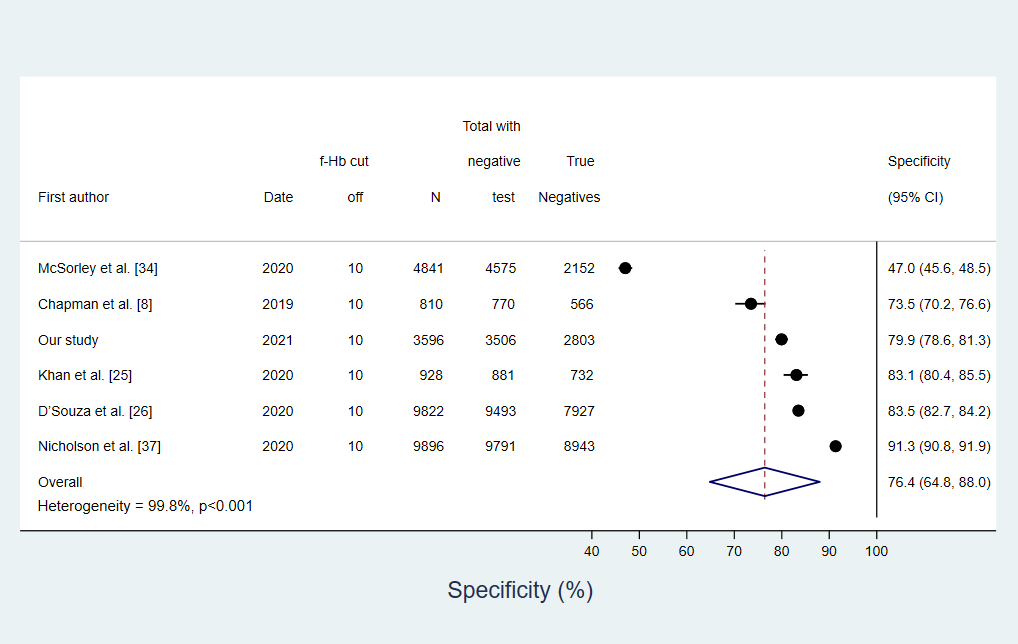


**Supplementary Figure 7.** Meta-analyses of 6 recent studies conducted in the UK, providing the combined CRC miss rate and specificity using a cut-off of 10 f-Hb for all studies. Excluding McSorley et al (low specificity), the pooled specificity is 82.4 (95% CI 76.9-87.8). The OC Sensor for the FIT assay was used in Chapman et al^8^ and our own study, and all others used HM-JACKarc. 877 CRC cases (N=total; FN = missed cancers/ false negatives).


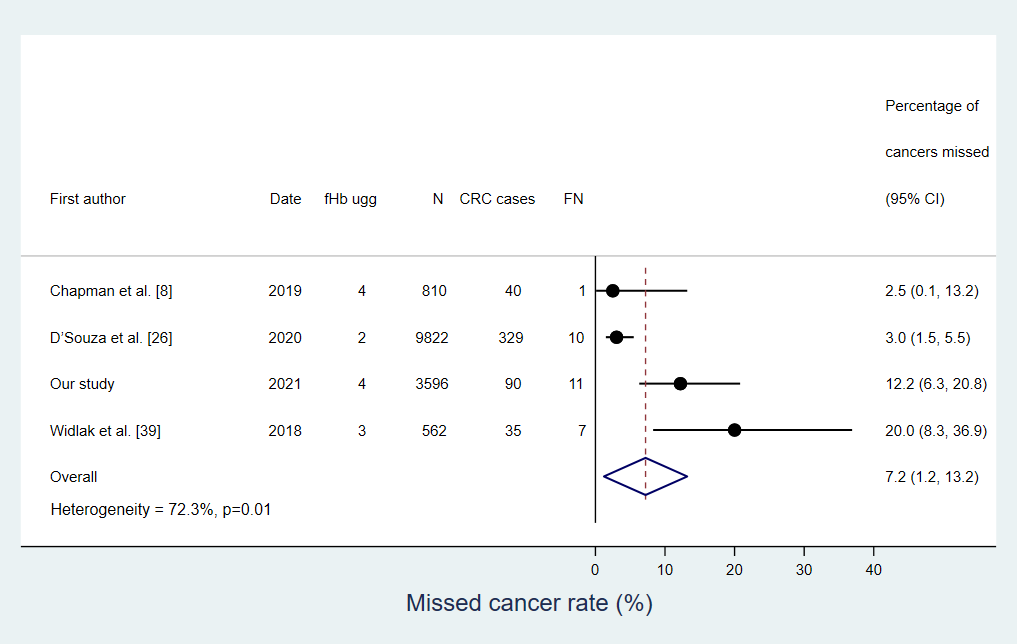

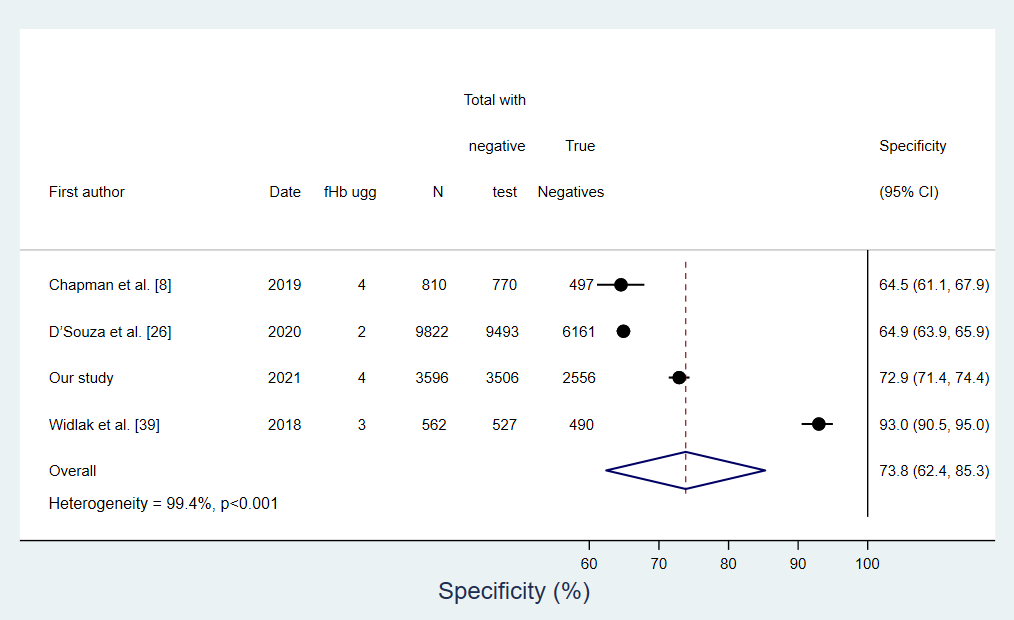


**Supplementary Figure 8.** Meta-analyses of 4 recent studies conducted in the UK, providing the combined CRC miss rate and specificity using a cut-off of f-Hb <4 for all studies. The OC Sensor for the FIT assay was used in Chapman et al^8^ and our own study, and all others used HM-JACKarc. 494 CRC cases (N=total; FN = false negatives, ie missed cancers).
